# Effectiveness of physical training for self-employed persons with musculoskeletal disorders: a randomized controlled trial

**DOI:** 10.1186/1471-2458-9-200

**Published:** 2009-06-23

**Authors:** Judith Heinrich, Johannes R Anema, Ernest MM de Vroome, Birgitte M Blatter

**Affiliations:** 1 TNO Quality of Life, Polarisavenue 151, PO Box 2130 AS Hoofddorp, The Netherlands; 2 Body@Work, Research Center Physical Activity, Work and Health TNO Vumc, Amsterdam, The Netherlands; 3 Department of Social Medicine, Institute for Research in Extramural Medicine, VU University Medical Center, Van der Boechorststraat 7, 1081 BT Amsterdam, The Netherlands; 4 Research Center for Insurance Medicine AMC-UWV-VUmc, Amsterdam, the Netherlands

## Abstract

**Background:**

Despite the fact that the population of self-employed persons is still growing and at risk for long term disability due to a number of risk factors, there is still a lack of information on the effectiveness of interventions for this specific group.

**Methods:**

To determine the effectiveness of physical training without a cognitive behavioral component and workplace specific exercises (PT) and physical training with a cognitive behavioral component and workplace specific exercises (PTCBWE), we conducted a pragmatic Randomized Controlled Trial, stratified into two groups. Self-employed persons with a new work disability claim because of musculoskeletal disorders were randomized to PT (n = 53) or PTCBWE (n = 76), or to a corresponding usual care group (n = 50 and n = 75 respectively). Both types of training consisted of cardiovascular training, strengthening, relaxation and posture exercises and took place two or three times a week, for 1–1.5 hours, during three months, also if someone had already returned to work full-time. The primary outcome measure was claim duration (in days) during 12 months follow-up. Pain severity and functional status were secondary outcome measures. All data were assessed at baseline and at 6 and 12 months follow-up. The data with regard to claim duration were analyzed by survival analysis and Cox regression analysis. Secondary outcome measures were analyzed by means of linear regression analysis.

**Results:**

After 12 months of follow-up there was no difference in claim duration between PT and usual care (Hazard Ratio 0.7; 95%CI, 0.4–1.1; p = 0.12) or PTCBWE and usual care (Hazard Ratio 0.9; 95%CI, 0.6–1.4; p = 0.72). Both types of physical training and usual care improved in pain and functional status over time, but there was only a statistically significant difference in favor of PT on pain improvement at 6 months.

**Conclusion:**

In this study, physical training with and without a cognitive behavioral component and workplace specific exercises for self-employed persons with musculoskeletal disorders was not shown to be effective on claim duration, pain severity and functional status at 12 months follow-up.

**Trial registration:**

Current Controlled Trials ISRCTN67766245.

## Background

### Musculoskeletal disorders

Musculoskeletal disorders (MSDs), of which low back pain (LBP) comprises the larger part, represent a considerable public health problem in Western industrialized societies. Almost 75% of the general population in the Netherlands reported any musculoskeletal pain during the past 12 months[1]. MSDs show an episodic pattern and in most cases the complaints improve spontaneously over time, without medical intervention or sick leave[1]. Although only a small percentage of people develop chronic pain, the consequences such as limitations in daily life, sickness absence, work disability and health care costs, account for high economic costs[2].

### Interventions

Interventions are predominantly aimed at prevention of chronic complaints, prevention of sickness absence, and return-to-work (RTW). Within the context of LBP, many interventions aimed at RTW include physical exercise programs. Studies which evaluate these interventions concentrate mainly on the effects of specific exercises and to a lesser extent on the effects of cardiovascular training; evaluations of a multidisciplinary approach are rare[3]. For example, Staal et al., (2004) evaluated the effect of graded activity for low back pain among employees in occupational health on sick leave, functional status and pain. With the biopsychosocial model as the underlying concept, the graded activity intervention is based on a physical exercise program, using operant-conditioning behavioural principles, to stimulate a rapid return to work. The results showed that graded activity was more effective than usual care in reducing the number of sick leave days in the first period of sick leave after randomization[4]. In general, the results of comparable studies show that employees following these kind of interventions return to work faster, indicate a greater reduction in pain intensity and experience less recurrences[3]. However, interpretation of the body of studies on physical exercise programs is difficult since they differ greatly with regard to the content-related and contextual factors, the target population, the definition of outcome measures and the follow-up period. Furthermore, in contrast with the increasing knowledge on the effects of physical exercise programs, little is known about the actual contents and the underlying concepts of working mechanisms because it is hard to identify which element of a program (physical training, cognitive behavioural component or workplace specific exercises) is responsible for which of the observed changes in outcome[5]. Among the studies reviewed by Staal et al. [5] only Lindström [6] reported the effects of the physical exercises on physical fitness variables, following graded activity intervention including a cognitive behavioural and workplace component. The participants in the graded activity group did improve significantly better in cardiovascular fitness at 1-year follow-up compared with the control group that received traditional care [6]. In general, there is some evidence that physical exercises as a component of RTW interventions do influence variables regarding physical fitness[5]. To summarize: conclusive scientific evidence concerning physical exercise programs with and without cognitive behavioural component and workplace specific exercises is still lacking.

### Self-employed persons

In addition, almost all studies focus on employees and LBP, although in the Netherlands 14% of the working population is self-employed and their number is still growing[7]. Furthermore, shoulder and neck pain are slightly less prevalent than LBP[1]. As far as we know, there is only one study performed regarding the evaluation of an early intervention with an integrated approach for self-employed persons who are disabled because of LBP[8]. The results of this study were positive. There is surprisingly little information available regarding physical exercise programs with and without a cognitive behavioral component and workplace specific exercises for self-employed persons[3], even though some groups of self-employed persons, especially agricultural workers, experience many MSDs[9]. It is known that self-employed persons have a longer claim duration than employees[8,10]. There are also some other differences between employees and self-employed persons which may influence the prognosis and effectiveness of an intervention[10]. For instance, self-employed persons are characterized by high levels of intrinsic motivation to work, long working hours, job control, job insecurity, work demands, decision latitude, type-A personality, and low levels of social support in their work[11]. Moreover, self-employed persons often only stop working when their complaints are already advanced and since their working weeks are extremely long, time to recover is limited. These specific characteristics specify the need for further research among self-employed persons. Therefore, the purpose of this pragmatic study is to determine the effectiveness of physical training based on the biopsychosocial model with and without a cognitive behavioural component and workplace specific exercises for self-employed persons with MSDs on claim duration, pain severity and functional status.

## Methods

In the Netherlands, only between 1998 and 2004 there was a social insurance system for self-employed persons still on sick leave after a deferred period of one year. This social insurance system provided compensation for loss of income to a maximum of 70% of the individual income but also limited to 70% of the statutory minimum income. The financial gap regarding the first year of sick leave and the difference between maximum compensation and normal income could be bridged by an insurance policy. Since 2004 this social insurance system is stopped and nowadays self-employed persons are no longer legally insured. It is the responsibility of every self-employed person him or herself to arrange an individual insurance, which provides supplementary compensation for loss of income if they are unable to work due to illness or an accident. A person can choose between different insured daily compensation amounts and deferred periods which influences the premiums. Such insurance is not compulsory, only 50% of the self-employed persons in the Netherlands has arranged an income insurance.

### Physical Training *without *a cognitive behavioural component and workplace specific exercises (referred to as PT)

The physical training took place two or three times a week, for 1–1.5 hours, during three months, also if someone had already fully returned to work again. It consisted of cardiovascular training, strengthening, relaxation exercises and posture exercises. Because the physical training exists of multiple components, a general level of intensity, which represents the whole training, can not be described. The level of intensity for every component was decided during an intake (as described below). At the start of the training, the level of intensity was just below the level of the intake. During the training, the level of intensity gradually increased. For every participant an individual level of intensity and gradually increase schedule was determined. The training was predominantly given by physiotherapists or trainers with a comparable background. Since this intervention had already been used by the physiotherapists or trainers for several years, they did not need training before this study. Before the start of the training, the physiotherapist or trainer inquired about the participant's medical history and completed a brief physical examination. Information about background, working conditions, functional status and pain was gathered by an intake questionnaire. The purpose of this intake was to make sure that there were no contra-indications (e.g. specific treatment needed) and to check the willingness to participate. Participants could also consult their general practitioners during the training period. Although the training was given in groups of 6–8 participants, everybody carried out individually tailored exercises based upon intake information next to the general exercises. During the course of the training this exercise schedule could be adjusted. The primary goal of physical training was to improve physical capacity, to learn to cope with complaints and to stimulate correct postures/movements. The overall goal of physical training was earlier and long-lasting return to work.

### Physical Training *with *a Cognitive Behavioural component and Workplace specific Exercises (referred to as PTCBWE)

The physical training component did not differ from the intervention described above regarding PT except the fact that during PTCBWE co-intervention (e.g. physiotherapy) was not allowed. An important aim of the added cognitive-behavioral component was to detect dysfunctional thinking habits and to change those thinking habits into a more realistic or functional way of thinking (e.g. reconceptualisation of pain). Participants were encouraged to focus on the functional level they could achieve and not on the pain[12]. This component of the training took about half an hour in every training session. The workplace specific exercises were developed after a workplace visit supported by video recording. This video was analyzed and discussed with the participants. Afterwards, the participants were stimulated to train the individually based techniques in their own workplace.

### Study design and population

To determine the effectiveness of physical training with and without a cognitive behavioral component and workplace specific exercises (PT and PTCBWE), we conducted a Randomized Controlled Trial (RCT) stratified in two groups. One group focused on the effectiveness of physical training without a cognitive behavioral component and workplace specific exercises (PT), the other group focused on the effectiveness of physical training with a cognitive behavioral component and workplace specific exercises (PTCBWE). For both types of physical training a corresponding control group was formed. Outcome measures in this study were claim duration, pain severity and functional status. The study population consisted of self-employed persons insured by a large Dutch insurance company that provides work disability insurances. The source population (n = 54.000) consisted of self-employed persons in all parts of the Netherlands, predominantly agricultural workers but also other occupations. All persons with a new claim episode were invited to take part in this study if they met the following inclusion criteria: (1) nonspecific musculoskeletal disorder (2) unable to fulfill his/her job for more than 25% according to a medical assessment (3) claim duration between 1 day and 8 weeks (including a deferred period) and (4) the musculoskeletal disorder can potentially be treated with physical training (according to the previous course of the disability, the patients' need and the assessment of the claim reviewer). When someone met the inclusion criteria he or she received written and oral information about the study purpose and procedures and was enrolled after giving informed consent. The Medical Ethics Committee of the University Medical Center in Leiden, the Netherlands, approved the study design, protocols, procedures and informed consent procedure.

### Treatment allocation and blinding

Assignment to PT or PTCBWE and usual care took place after completion of the baseline questionnaire and informed consent and was performed on individual level on the basis of a chance (random) process. Due to this procedure, every individual had a chance of 50% to be assigned to either physical training or usual care. Two boxes (for PT and PTCBWE separately) with non-transparent, sealed envelopes were prepared by a researcher who was not involved in enrolling participants or assigning participants to their groups. The envelopes contained papers indicating PT or PTCBWE or usual care and were sequentially opened. The treatment allocation was made known to the participant by a research assistant. The primary outcome measure, claim duration (in days), was derived from databases from the insurance company. The end date of the claim and the level of work disability were determined by a claim reviewer, the medical advisor and a general practitioner hired by the insurance company. None of these persons could totally be blinded. However, they did not have benefits from physical training. Although blinding of the other, self-reported, outcome measurements (pain severity and functional status) during follow-up was not possible, there was no direct influence by the researchers or treating professionals because all questionnaires were mailed to the participants.

### Usual care

The participants, who were allocated to the usual care group, received the usual guidance by their general practitioner according to the guidelines of the Dutch College of General Practice for musculoskeletal disorders. A detailed description of this usual care treatment is complicated to give beforehand, since participants with MSDs on different locations were included. In addition, the general practitioner will only propose a specific treatment after anamnesis. For participants with low back pain, e.g. this could result in physiotherapy treatment according to the Low Back Pain Guideline of the Royal Dutch College for Physiotherapy[13]. This guideline recommends giving adequate information, advising to stay active and providing exercise therapy with a behavioural approach. As the guideline physiotherapy is not a protocol, the number of sessions varies per patient. In daily practice patients receive on average 9 treatment sessions and the average duration of treatment is 6 weeks[14].

### Outcome measures and data-collection

The primary outcome measure, claim duration (in days), was defined as follows: the number of days the participant received work disability compensation between the moment of randomization and 12 months later. This outcome was determined by counting the number of calendar days from randomization, till the end date of the claim period and was not adjusted for the level of work disability (gross duration). Secondly, net duration was calculated by adjusting gross duration for the level of work disability (net duration). The end of the claim period was defined as less than 25% work disability according to medical assessment with a minimum duration of 4 weeks. This means that recurrences of work loss due to the same disorder within 4 weeks of the end of the claim were considered as belonging to the same first continuous claim period. In addition, we also calculated the number of participants in both groups with additional claim recurrences (> 4 weeks after the first episode) due to the same disorder during the entire 12 months of follow-up. Data on claim duration and level of work disability were continuously collected by means of the electronic records of the insurance company. Pain severity and functional status were defined as secondary outcome measures. Pain severity was evaluated by two questions (pain in the previous 6 months and pain at this moment) on an 11-point numerical scale ranging from 0 (no pain) to 10 (very severe pain) with clearly and equally spaced intervals[15]. Functional status of the participants was assessed by several question from the Neck Pain Disability Index (NPDI) and the Quebec Back Pain Disability Scale (QBPDS), both leading to a sum score between 0–100[16,17]. Previous studies showed that the Dutch translation of the QBPDS and also of the NPDI proved to be a reliable and valid instrument[16,18]. In this study both modules were used, because the study population existed of persons with MSDs on diverse locations. For both pain severity and functional status, the outcome measure was defined as the mean score at baseline minus the mean score at follow-up. Finally, data were collected on possible prognostic factors such as history of complaints, own expectation on return-to-work and claim duration before randomization. Prognostic factors were derived from the literature[8,19-22]. Data collection for secondary outcome measures and prognostic factors took place at baseline before randomization, and at 6 and 12 months follow-up.

### Statistical analyses

To examine the success of randomization, statistical tests (Student's t-test, Chi-square test and Mann-Whitney U test) were used to compare baseline characteristics. The data with regard to the new claim period were analyzed by survival analysis. Kaplan Meier analysis was used to describe the distribution of the claim duration of both study groups. These distributions of the groups were compared and the difference was tested by means of the log-rank test. With Cox regression analysis, Hazard Ratios (HR) (and 95% confidence intervals) including correction for two possible confounders (general health and duration of disability before randomization) were calculated. The primary outcome for the Cox regression analyses was claim duration (in days) between randomization and 12 months follow-up. The Cox proportional hazard assumption was taken into account by analyzing the first 6 months of follow-up separately from the total of 12 months of follow-up. Pain severity and functional status were analyzed by means of regression analysis. Prognostic factors from the literature were all treated as potential confounders and tested for collinearity[8,19-22]. In the final model two variables with a clinically relevant difference between intervention and control group were included as confounders (history of complaints and partial or full work disability at baseline). Because the study population was relatively small, a clinically relevant difference was defined as at least 1/5 of the value over both treatment groups[23,24].

At first all analyses were performed according to the intention-to-treat principle. Secondly, to examine the effects of the treatment solely among those who actually received the treatment, we conducted per-protocol analyses excluding all participants from the analyses who were not treated according to the protocol (results not shown in tables). A time-dependent covariate was added in the per-protocol analysis to adjust for the claim duration between randomization and treatment. For the physical training groups (PT and PTCBWE) we included only participants in this per protocol analysis who had a positive intake, who started with physical training and had a compliance rate of at least 80%. The intake was positively evaluated when the participants still experience symptoms and were unable to fulfill their job for more than 25%, when they were motivated to start physical training and when the distance to the training center was not a barrier. The 80% limit was somewhat arbitrary chosen because ideally we wanted to include only participants with 100% compliance. However, holding on to this 100% limit yielded very small groups. Therefore, we decided to lower this to 80%. For the usual care group we included only participants who did not start physical training as evaluated in this study. To be able to demonstrate an effect of at least a difference of 20 days of claim duration, the sample size calculations showed that a target sample of 75 participants in each group within each type of physical training was needed (power of 0.80 and two-tailed significance level of 0.05). Values of p < 0.05 were considered statistically significant. All analyses were done with SPSS for Windows 14.0 (SPSS inc., IL, USA).

## Results

### Study population

**Figure 1:**
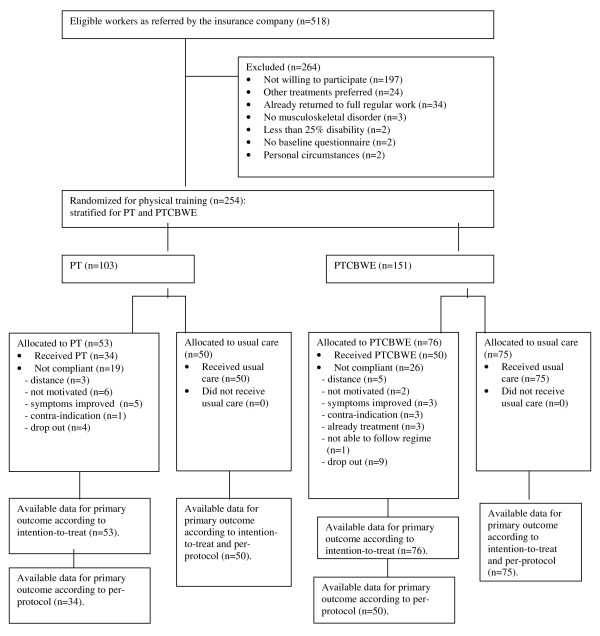
**Flow diagram describing the progress of participants through the phases of the trial. PT refers to physical training without a cognitive behavioral component and workplace specific exercises. PTCBWE refers to physical training with a cognitive behavioral component and workplace specific exercises**. *This figure presents the flow of (non-)participants from the inclusion period of the study till the end of follow-up. The number of participants included in each arm of the trial is the key information of this figure. Moreover, also information about exclusion of workers is given. Finally, this figure informs about the reasons why participants were not compliant to their treatment allocation*.

From November 2004 till December 2006 518 self-employed persons were referred to the research assistant. Information regarding the flow of participants through the trial is presented in Figure 1. There were many self-employed persons who were not willing to participate (197 of 518). Nevertheless, a detailed non-response analysis of anonymous data on claim duration and level of work disability showed no differences between participants and non-participants. Data on claim duration and work disability level were collected for all participants. The response on secondary outcome measures was 60% (6 months) and 65% (12 months) for PT and 68% and 64% for PTCBWE respectively. Table 1 shows the baseline characteristics and the baseline values of the outcome measures for both groups. There was only a statistically significant difference for history of complaints between the intervention and control group regarding PT.

**Table 1:** Baseline values of outcome measures and potential prognostic variables

	Physical training (PT)		Physical training with a cognitive behavioral component and workplace specific exercises (PTCBWE)	
	
	Intervention (n = 53)	Control (n = 50)	Intervention (n = 76)	Control (n = 75)
Age (yr) (mean (SD))	46 (7.1)	45 (8.4)	45 (6.6)	45 (7.1)
Gender (male) (n (%))	49 (93)	46 (96)	69 (91)	70 (93)
Industry (agriculture) (n (%))	27 (51)	27 (56)	48 (63)	49 (65)
Location of complaints (low back pain) (n (%))	25 (47)	21 (42)	39 (51)	34 (45)
Expectation of participants on return to work (n (%))				
- a few days till one month	3 (6)	3 (6)	16 (22)	12 (16)
- a couple of months	22 (42)	18 (38)	17 (23)	16 (22)
- no idea	25 (48)	25 (52)	35 (47)	45 (62)
- never	2 (4)	2 (4)	6 (8)	0 (0)
Full disability at randomization (n (%))	13 (25)	13 (26)	23 (30)	18 (24)
History of complaints (yes) (n (%))	15 (28)	34 (71)*	41 (54)	44 (59)
Good general health (n (%))	25 (47)	28 (58)	51 (67)	40 (53)
Insured daily compensation (euros) (mean (SD))	66 (20)	66 (34)	74 (25)	70 (23)
Duration (weeks) of disability before randomization (median (IQR))	8 (6–13)	9 (6–16)	10 (5–14)	8 (5–14)
Pain severity (0–10)† (mean (SD))	5.9 (1.2)	5.8 (1.9)	5.9 (1.4)	5.6 (1.7)
Functional status (NPDI, 0–100)† (mean (SD))	37 (14)	37 (14)	38 (13)	35 (14)
Functional status (QBPDS, 0–100)† (mean (SD))	34 (17)	36 (17)	35 (16)	35 (17)

* p < 0.05

† A higher score means a higher level of pain or functional restrictions.

IQR indicates interquartile range (25th – 75th percentile).

NPDI indicates the Neck Pain Disability Index.

QBPDS indicates the Quebec Back Pain Disability Scale.

Differences between the study groups concerning continuous and normally distributed outcome measures were tested with the Student's t-test.

Differences between the study groups concerning dichotomous or ordinal outcome measures were tested with the Chi-square test. In addition the adjusted standardized residual was determined.

Differences between the study groups concerning continuous but skewed distributed outcome measures were tested with the non-parametric Mann-Whitney U test (only duration of disability before randomization).

### Compliance to treatment and co-intervention

The duration of physical training without a cognitive behavioral component and workplace specific exercises (PT) was 18 weeks (median) and started at 5 weeks (median) after randomization. The duration of physical training with a cognitive behavioral component and workplace specific exercises (PTCBWE) was 14 weeks (median) and started at 4 weeks (median) after randomization. Nineteen participants were not compliant to the protocol of PT (for reasons, see Figure 1), leaving 34 participants in the intervention group for the per-protocol analyses. For PTCBWE, 50 out of 76 participants were compliant to the protocol. In addition to PT, 14 (42%) respondents indicated another treatment, predominantly physiotherapy according to the Low Back Pain Guideline of the Royal Dutch College for Physiotherapy (see section 'usual care')[13]. Although co-intervention was not allowed during PTCBWE, 16 (31%) respondents confirmed that they received some other treatment, mainly physiotherapy. In the first six months of follow-up 85% of the respondents in both usual care groups reported treatment for their complaints, predominantly physiotherapy (63%). Additionally, 43% of these respondents reported that their treatment contained some training or sporting activities.

### Claim duration results for physical training (PT)

**Figure 2:**
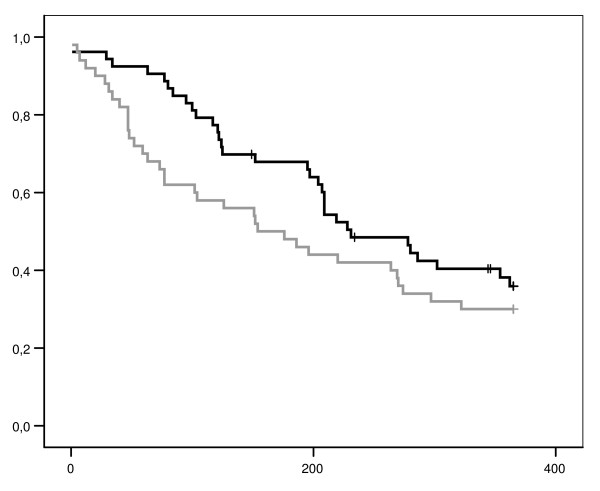
**Kaplan-Meier curves of claim duration for physical training without a cognitive behavioral component and workplace specific exercises (PT) and usual care**. x-axis: claim duration (days) since randomization. y-axis: proportion participants with a claim. dark line above: physical training (PT). grey line beneath: usual care. + censored. *This figure presents the survival curve (Kaplan-Meier) of claim duration within the group participants following PT (black line) and the corresponding group of participants following usual care (grey line). The Log-Rank test indicated p = 0.18, suggesting no difference between both groups in claim duration (in days) after 12 months follow-up (primary outcome measure)*.

In the first 6 months of follow-up there was a statistically significant difference in claim duration, but in favor of the usual care group. The median claim duration (gross) was 181 in the PT group and 153 in the usual care group (log-rank test; p = 0.03) (Figure 2 and Table 2). After adjusting for possible confounders this difference remained statistically significant; the Hazard Ratio (HR) was 0.5 (95% CI 0.3 – 0.9; p = 0.03) in favor of the usual care group. When the analyses were performed according to the per-protocol principle including a time-dependent covariate (for time between randomization and treatment), the HR lowered to 0.4 (95% CI 0.2 – 0.9; p = 0.03) for the period after physical training had started.

**Table 2:** Results of the univariate and multivariate survival analyses regarding claim duration (days)

			Univariate analyses		Adjusted Hazard Ratios (95% confidence interval) for return to work (Cox regression analyses) †	
			
			Claim duration (days) (median (IQR))*	Log rank (p)	HR	p-value
6 months	PT (n = 101)	Intervention	181 (119 – 184)			
		Control	153 (48 – 181)	0.03	0.5 (0.3 – 0.9)	0.03
	PTCBWE (n = 153)	Intervention	133 (70 – 183)			
		Control	137 (48 – 181)	0.60	0.8 (0.5–1.3)	0.43
12 months	PT (n = 101)	Intervention	228 (122 – 365)			
		Control	165 (48 – 365)	0.18	0.7 (0.4 – 1.1)	0.12
	PTCBWE (n = 153)	Intervention	148 (75 – 343)			
		Control	137 (48 – 365)	0.95	0.9 (0.6 – 1.4)	0.72

* Gross duration in days.

IQR indicates interquartile range (25th – 75th percentile).

† Adjusted for general health and duration of disability before randomization.

PT indicates physical training without a cognitive behavioral component and workplace specific exercises. PTCBWE indicates physical training with a cognitive behavioral component and workplace specific exercises.

In the univariate analyses Cox regression was used to determine the difference in claim duration in days between randomization and 6 and 12 months follow-up (primary outcome measure) among both study groups (main exposure). The control group (participants not randomized for physical training) served as the reference category. The multivariate analyses were the same but with a correction for two possible confounders: 'general health' and 'duration of disability before randomization'.

After 12 months the median claim duration (gross) was 228 in the PT group and 165 days in the usual care group (log-rank test; p = 0.18). Adjusting for possible confounders did not change these results (HR 0.7 95% CI 0.4 – 1.1; p = 0.12), neither did the per-protocol analyses. Since only a few participants experienced recurrent complaints, the results concerning recurrences are not presented. For both the short and the long term follow-up, the results did not alter when net duration was used as outcome measure.

### Claim duration results for physical training with a cognitive behavioral component and workplace specific exercises (PTCBWE)

**Figure 3:**
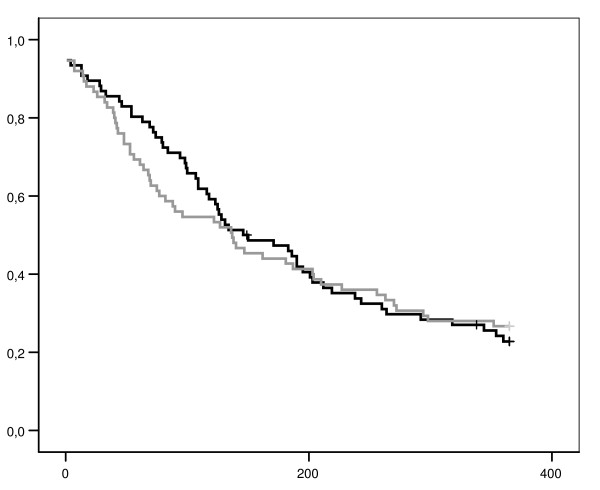
**Kaplan-Meier curves of claim duration for physical training with a cognitive behavioral component and workplaces specific exercises (PTCBWE) and usual care**. x-axis: claim duration (days) since randomization. y-axis: proportion participants with a claim. dark line above: physical training (PTCBWE). grey line beneath: usual care. + censored. *This figure presents the survival curve (Kaplan-Meier) of claim duration within the group participants following PTCBWE (black line) and the corresponding group of participants following usual care (grey line). The Log-Rank test indicated p = 0.95, suggesting no difference between both groups in claim duration (in days) after 12 months follow-up (primary outcome measure)*.

After 6 months, the median claim duration (gross) was 133 in the PTCBWE group and 137 in the usual care group (log-rank test; p = 0.60) (Figure 3), adjusting for possible confounders did not change these results (Table 2). When the analyses were performed according to the per-protocol principle including a time-dependent covariate (for time between randomization and treatment), the HR remained the same (HR 0.8 95% CI 0.5 – 1.3; p = 0.33).

The results of 12 months follow-up showed only a slight increase in claim duration for the intervention group (gross duration is 148 days), not for the control group (gross duration is 137 days). For the full 12 months follow-up HR was 0.9 (95% CI 0.6 – 1.4; p = 0.72). According to the per-protocol analyses both groups were even more similar at 12 months (HR 1.0 95% CI 0.7 – 1.6; p = 0.95). Since only a few participants experienced recurrent complaints, the results concerning recurrences are not presented. For both the short and the long term follow-up, the results did not alter when net duration was used as outcome measure.

### Results on pain and functional status for physical training (PT)

After 6 months the difference in mean decrease in pain was 1.4 points (scale 0–10)(95% CI 0.4 – 2.5) in favor of the PT group (p = 0.01) (Table 3). However, according to the per-protocol analyses this differences was only 0.7 points (p = 0.21). Concerning functional status as measured with the NPDI, the difference in mean improvement was 4.8 points (scale 0–100)(95% CI -5.2 – 14.9) in favor of the PT group (p = 0.34). Following the per-protocol analyses this difference was slightly smaller. Functional status as measured with the QBPDS showed similar results. The difference was 2.4 points (scale 0–100) (95% CI -9.9 – 14.8) in favor of the PT group (p = 0.70), and remained nearly the same when performing the per-protocol analyses.

**Table 3:** Mean improvements in pain and functional status: baseline, 6 and 12 months

			Mean (SD) improvement			
			
			Intervention	Control	Between-group difference † (95% CI)	p-value
6 months	PT (n = 62)	Pain	2.0 (1.8)	0.7 (1.6)	1.4 (0.4 – 2.5) ‡	0.01
		Functional status NPDI	14 (21)	8 (18)	4.8 (-5.2 – 14.9)	0.34
		Functional status QBPDS	13 (24)	10 (21)	2.4 (-9.9 – 14.8)	0.70
	PTCBWE (n = 102)	Pain	1.5 (2.0)	0.8 (2.0)	0.7 (-0.1 – 1.5)	0.09
		Functional status NPDI	15 (16)	9 (15)	5.3 (-0.8 – 11.5)	0.09
		Functional status QBPDS	13 (20)	10 (14)	2.7 (-4.0 – 9.5)	0.43
12 months	PT (n = 67)	Pain	2.3 (2.1)	1.6 (2.8)	0.9 (-1.2 – 1.4)	0.90
		Functional status NPDI	17 (18)	13 (24)	-1.2 (-11.9 – 9.4)	0.82
		Functional status QBPDS	15 (20)	16 (26)	-5.8 (-17.7 – 6.2)	0.34
	PTCBWE (n = 96)	Pain	2.1 (2.1)	2.0 (2.4)	0.03 (-0.9 – 1.0)	0.95
		Functional status NPDI	16 (18)	15 (18)	0.8 (-6.3 – 7.9)	0.83
		Functional status QBPDS	14 (20)	14 (18)	-0.3 (-7.9 – 7.3)	0.94

PT indicates physical training without a cognitive behavioral component and workplace specific exercises. PTCBWE indicates physical training with a cognitive behavioral component and workplace specific exercises.

† Adjusted for history of complaints and partial or full work disability at baseline.

‡ The mean decrease in pain is 1.4 points higher in the physical training group than in the usual care group. Pain severity and functional status (secondary outcome measures) were analyzed by means of linear regression analyses. Randomization result was the independent variable in the analyses, and history of complaints and partial or full work disability at baseline were included as potential confounders.

After 12 months the mean decrease in pain was 0.9 (95% CI -1.2 – 1.4) points higher in the PT group (p = 0.90) (Table 3). However, according to the per-protocol analyses the difference was smaller and in favor of usual care. For both NPDI and QBPDS the mean improvement in functional status was in favor of usual care, although not statistically significant, and per-protocol analyses only showed minor changes in the same direction.

### Results on pain and functional status for physical training with a cognitive behavioral component and workplace specific exercises (PTCBWE)

After 6 months the mean decrease in pain was 0.7 points (scale 0–10)(95% CI -0.1 – 1.5) higher in the PTCBWE group compared to the usual care group (p = 0.09) (Table 3). This difference became stronger during per-protocol analyses. Functional status as measured with NPDI showed a difference of 5.3 points (scale 0–100)(95% CI -0.8 – 11.5) in favor of physical training, and this difference became larger and statistically significant during per-protocol analyses. The mean improvement of functional status as measured with the QBPDS was 2.7 points higher (scale 0–100) (95% CI -4.0 – 9.5) in the physical training group, but this difference was not statistically significant. Results did not change after per-protocol analyses.

After 12 months the mean decrease in pain was nearly the same in both groups (p = 0.95) (Table 3). Also, the improvement of functional status, both NPDI and QBPDS, was more or less the same between groups (p = 0.83 and p = 0.94 respectively). The results showed only minor changes during per-protocol analyses.

## Discussion

### Explanation of the results

There are two major aspects which could (partly) explain the results. A first explanation could be contamination between the intervention and control groups which resulted in a smaller than expected contrast between physical training and usual care. As mentioned in the results only 64% and respectively 66% of the participants randomized to PT and PTCBWE complied with the protocol. In addition, 30–40% of the participants allocated to PT or PTCBWE reported co-intervention, predominantly physiotherapy. Co-intervention was only allowed to PT, but will always reduce the contrast between physical training and usual care. Moreover, 43% of the participants receiving usual care reported that their treatment contained some training or sporting activities. However, it is uncertain to which extent training or sporting activities in usual care were comparable to the physical training evaluated in this study. In general, training or sporting activities are less intensive in frequency and duration (compared to PT and PTCBWE), there is less control on the compliance of participants and, most importantly, the focus is mainly on pain reduction instead of long-standing return-to-work. We tried to exclude participants in usual care reporting training or sporting activities during the per-protocol analyses, but unfortunately after this selection the control group became too small for further analyses. Secondly, it is known that participants might be less inclined to return to work during active treatment periods[4]. Since the evaluated intervention lasted on average three months, this aspect could also have reduced the effect of the intervention. Furthermore, some claim reviewers put the level of disability or the duration of the claim 'on hold' during the course of physical training, waiting until the training had ended. This process could be a result of an arrangement with the insured for compensation of the invested time, or because the claim reviewer only expects improvement after the training has finished. The handling of a claim by the insurance company could therefore have influenced the results regarding the claim duration in the group of physical training[8]. Another explanation could be that the baseline values regarding physical capacity and cardiovascular strength of this specific group, predominantly existing of agricultural workers, were very high. Although the training centers collect this kind of information during the intake, this information was not used as outcome measure since we focus on the perspective of the insurance company (claim duration as primary outcome measure). Nevertheless, the physiotherapists and trainers all indicate that agricultural workers at the intake show high strength capacity and to a lesser extent also aerobic capacity. Because the level of intensity is based on this intake information, we expected that every participant could improve within his own range. Finally, the different results between PT and PTCBWE may have been influenced by differences in training components since only PTCBWE contained a cognitive-behavioral component and workplace specific exercises.

### Comparison with other studies

First of all, we want to mention that the results of this study were difficult to compare with other studies in this field because of the specific self-employed population. Besides, the primary outcome measure of this study was claim duration while comparable studies by employees focus mostly on return-to-work. As pointed out earlier, the end of a claim or compensation period does not necessary equal full return-to-work[8]. Therefore, claim duration in the present study could only be interpreted as a proxy for time to full return-to-work. Our results are comparable with the results of other studies evaluating high-intensive interventions aimed at return-to-work [25-28]. In the study of Torstensen et al. (1998)[25] progressively graded medical exercise therapy is compared with conventional physiotherapy. They did not find any statistically significant difference between both groups. The comparability with the current study might be a result of the control group, since also in the current study more than half of the control population received conventional physiotherapy. The second study[26] focused on back schools and showed that only workers in the low-intensity back school returned to work faster compared to usual care. The comparison between high intensity back school and usual care resulted in a HR of 1.0. In the third study[27,28], graded activity delayed time to return-to-work. Two explanations given by the authors were a delay in the referral process and low compliance of the workers randomized to the graded activity intervention. All these studies with comparable results were pragmatic trials concentrated on effectiveness. On the contrary, in two more idealistic trials, graded activity seemed effective in time to return-to-work for workers on sick leave for eight weeks[6] or less[4]. These studies were performed in specialized in-company physiotherapy clinics by a limited number of physiotherapists and as a result there were fewer problems with implementation and compliance. Consequently, the effectiveness of an intervention may be influenced by the involvement of and the frequency and content of communication between employees, providers, treating physicians, occupational physicians and employers[29]. The insurance setting in the present study could therefore also be partly responsible for the results.

### Strengths of this study

A principal strength of this study is that, to our knowledge, this is to date one of the two RCT's that evaluated the effectiveness of an intervention within a study population of self-employed persons[11]. More attention should be focused on self-employed persons, since this group is relative large, still growing and a vulnerable population for long term work disability due to a number of risk factors. Another strength of this study is that all kinds of musculoskeletal disorders are included in the study. Although most persons experience LBP, as in our study, information about the effectiveness of interventions for other nonspecific MSDs is also necessary. Finally, both types of physical training (PT and PTCBWE) were already given by the physiotherapist or trainers for a couple of years, so they had long term experience in practice and their skills were not a problem. Along with this point we want to remark that our study focused on effectiveness instead of efficacy. Effectiveness refers to the impact of an intervention in the real world situation, whereas efficacy refers to the impact of an intervention in a clinical trial. Due to this pragmatic RCT design, the generalizability and usability of the results for practice is good, but, not all parameters could be controlled. As a result, there are also some limitations of this study.

### Limitations of this study

The first limitation concerns the relatively small sample that was obtained to evaluate physical training without a cognitive behavioral component and workplace specific exercises (PT) (103 instead of 150). Because of this limited sample size, the power to detect expected differences after 12 months follow-up may have been too small.

This relatively small sample might be a result of the large non-response (197 of 518) especially compared with similar studies among employees (4 of 150)[4] and (18 of 243)[26]. This may partly be explained by the specific situation of self-employed persons. In a previous study of self-employed persons 211 of 462 refused participation[11]. The other limitation is the relatively long duration between both the onset of the claim and randomization (median 8 weeks) and between randomization and the start of physical training (PT/PTCBWE) (median 4 weeks), whereas the target population of the training institutes exists of people with 3 to 12 weeks complaints and/or disability. Earlier studies described that the effectiveness of many disability management interventions and their optimal timing remains unknown[30]. Although several strategies explicitly focus on early interventions, the actual pattern of duration dependence has hardly been investigated. Knowing the probability patterns and duration dependence of different groups of workers in different settings can be helpful in making decisions in intervention strategies. In some cases, it may be preferable to intervene late instead of early[31]. Because of a lack of information about this in our study population it is not known whether the relatively late onset of physical training is in general positive or negative. Future research should focus on probability patterns and duration dependence since only with this information can be indicated which insurants would benefit most from the physical training and what the optimal timing of physical training would be.

## Conclusion

The aim of this study was to determine the effectiveness of physical training without a cognitive behavioral component and workplace specific exercises (PT) and the effectiveness of physical training with a cognitive behavioral component and workplace specific exercises (PTCBWE) for self-employed persons with MSDs in the Netherlands. After 6 months, PT was less effective on claim duration than usual care. However, PT was effective on improvement of pain severity. After 12 months, PT was not effective on claim duration compared to usual care. Concerning pain severity and functional status on the long term, no statistically significant differences were found for PT.

PTCBWE was not effective on claim duration, both on the short and the long term. Pain severity and functional status improved in both groups, but there were no statistically significant differences.

These conclusions are important given that the group of self-employed persons is relatively large, still growing and a vulnerable population for long term work disability due to a number of risk factors. More attention should be focused on research project within self-employed persons.

## Competing interests

The authors declare that they have no competing interests.

## Authors' contributions

JH: was involved in the conception and design of the study, carried out the data collection and statistical analysis, and drafted the manuscript. JRA: was involved in the conception and design of the study, gave structural advice concerning the statistical analysis and delivered an important intellectual content regarding the revision of manuscripts. EMMV: performed the data conformation, was involved in the statistical analysis and delivered critical improvements to the manuscript. BMB: was involved in the conception and design of the study, coordinated the study, gave structural advice concerning the statistical analysis and helped to draft the manuscript. All authors read and approved the final manuscript.

## Pre-publication history

The pre-publication history for this paper can be accessed here:


